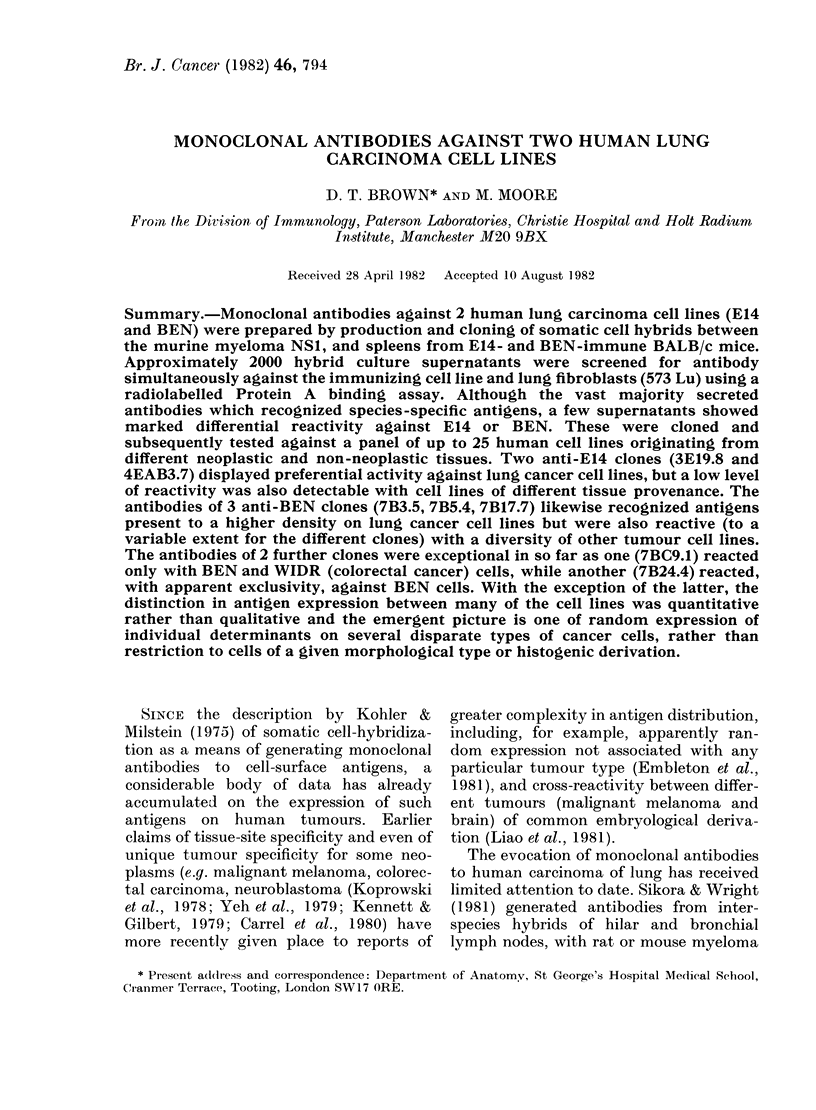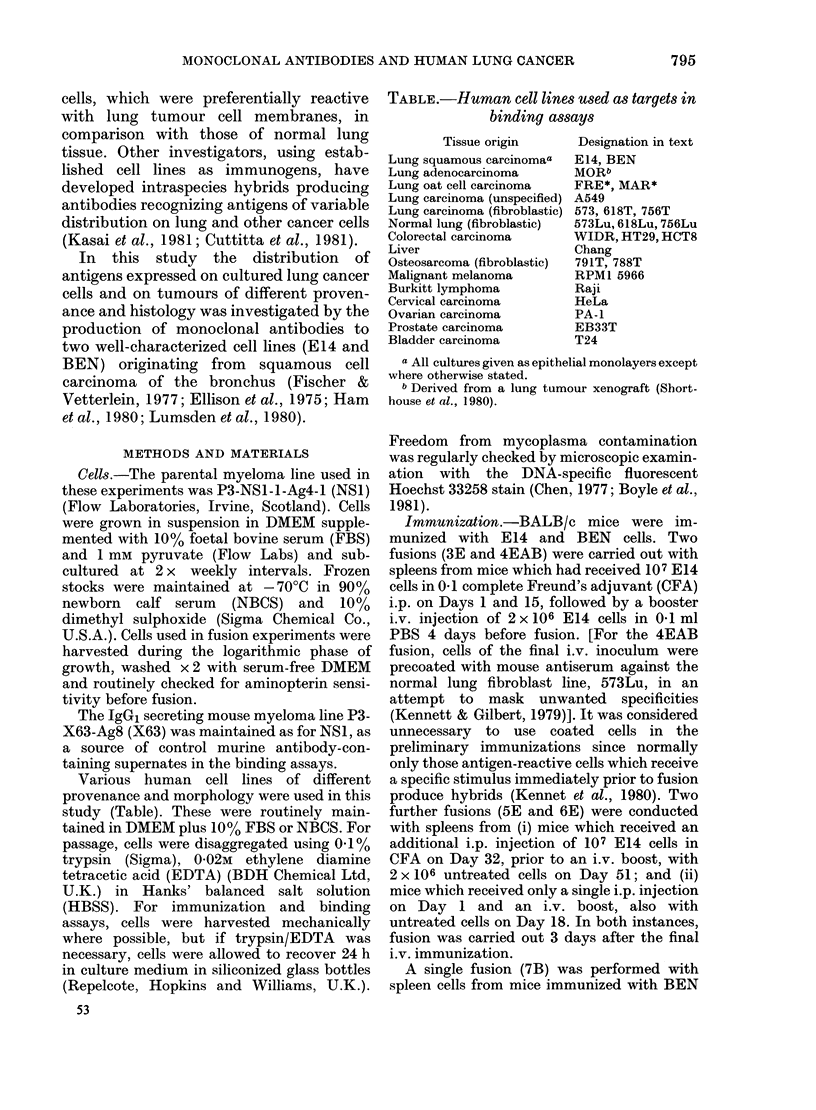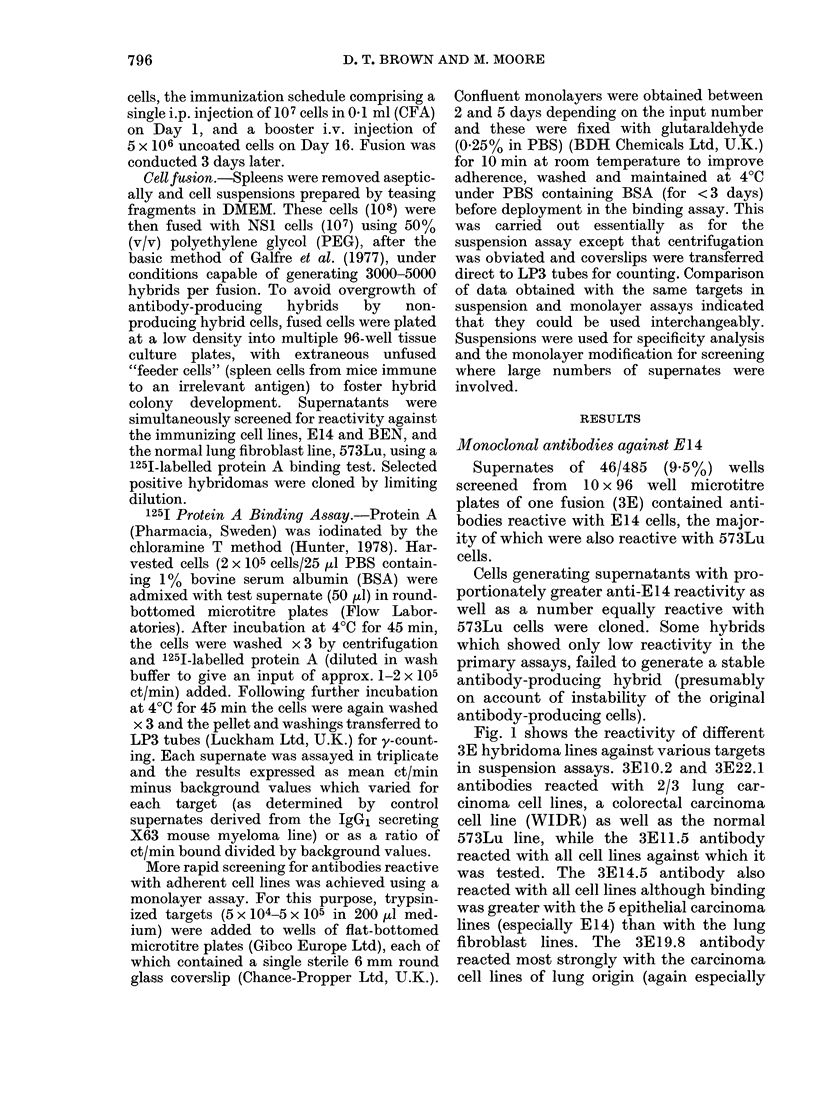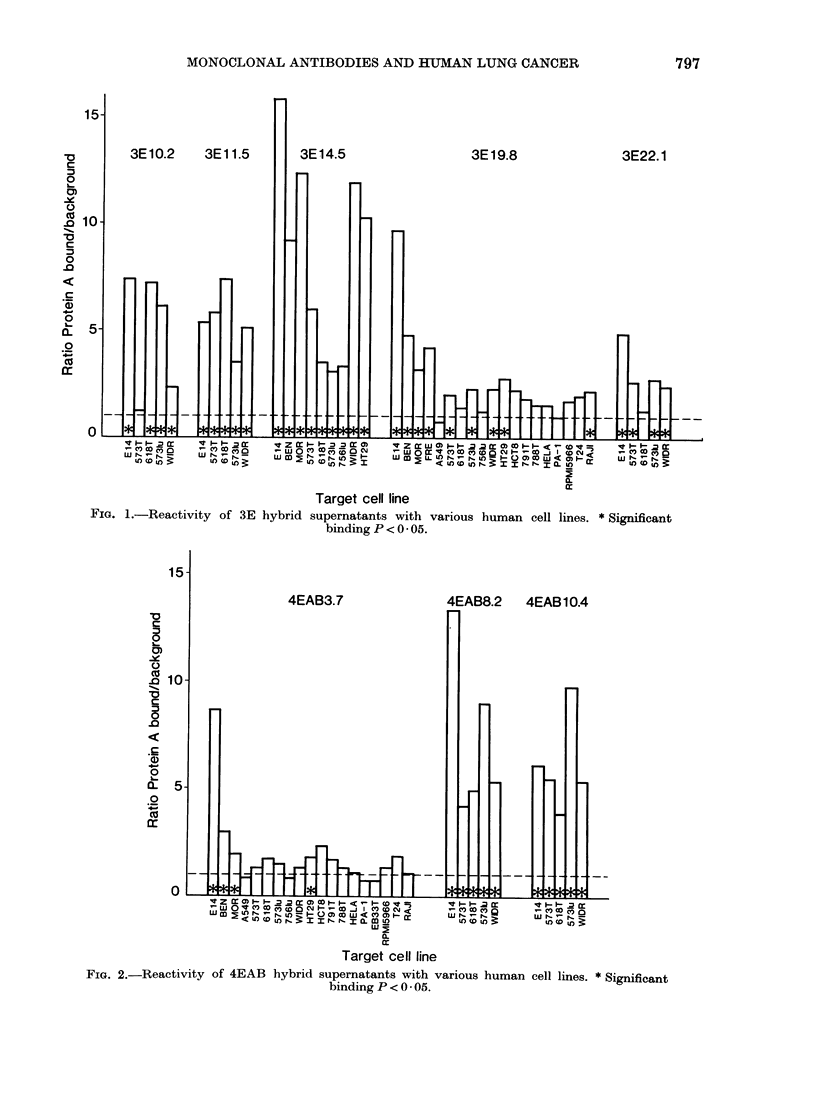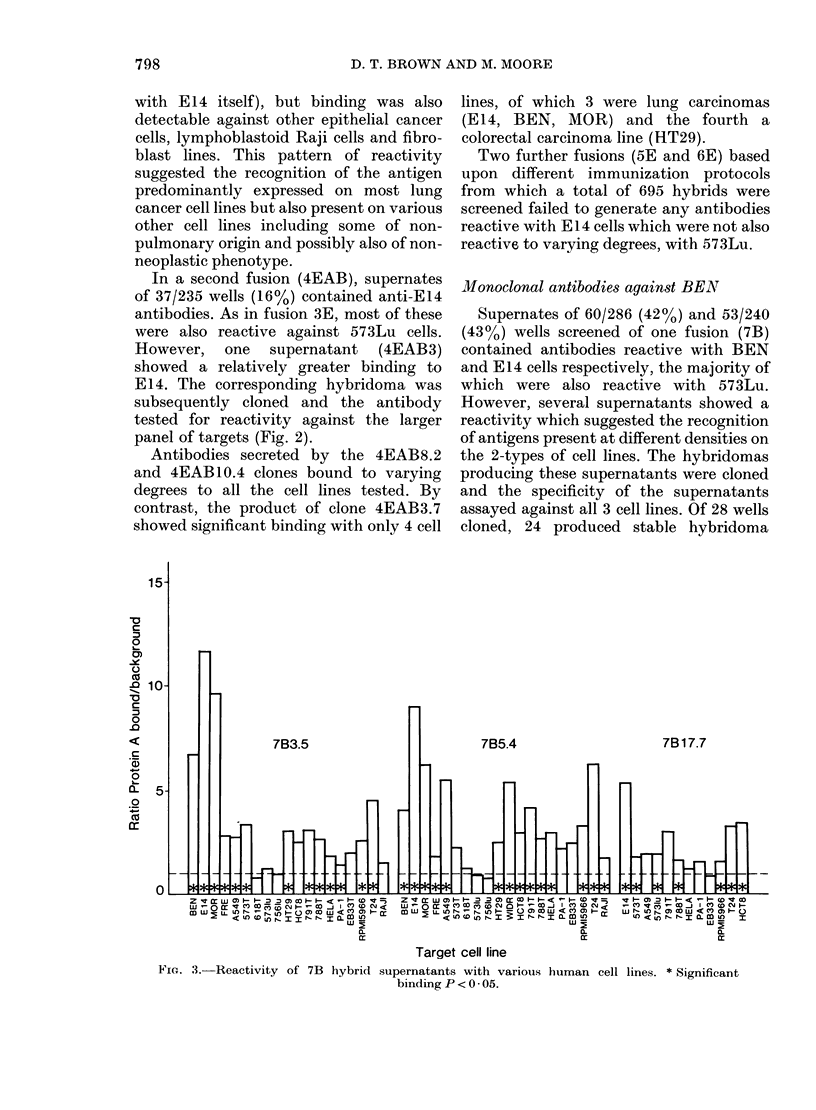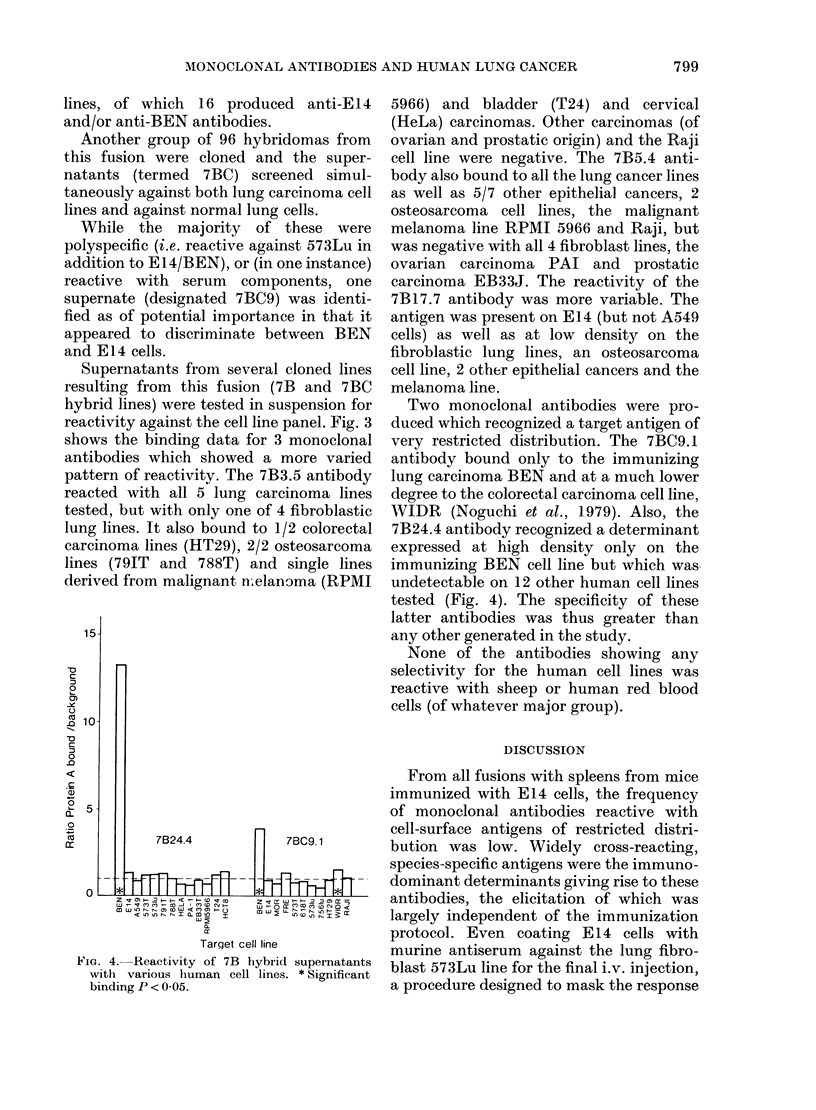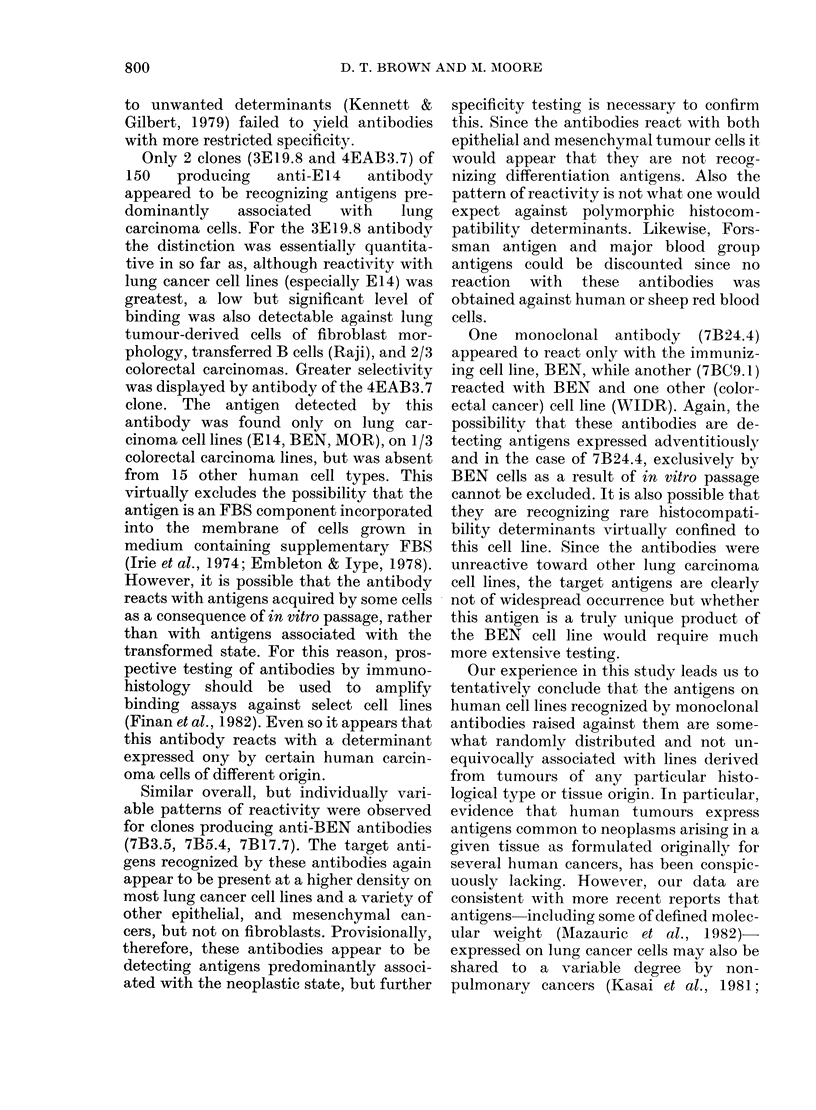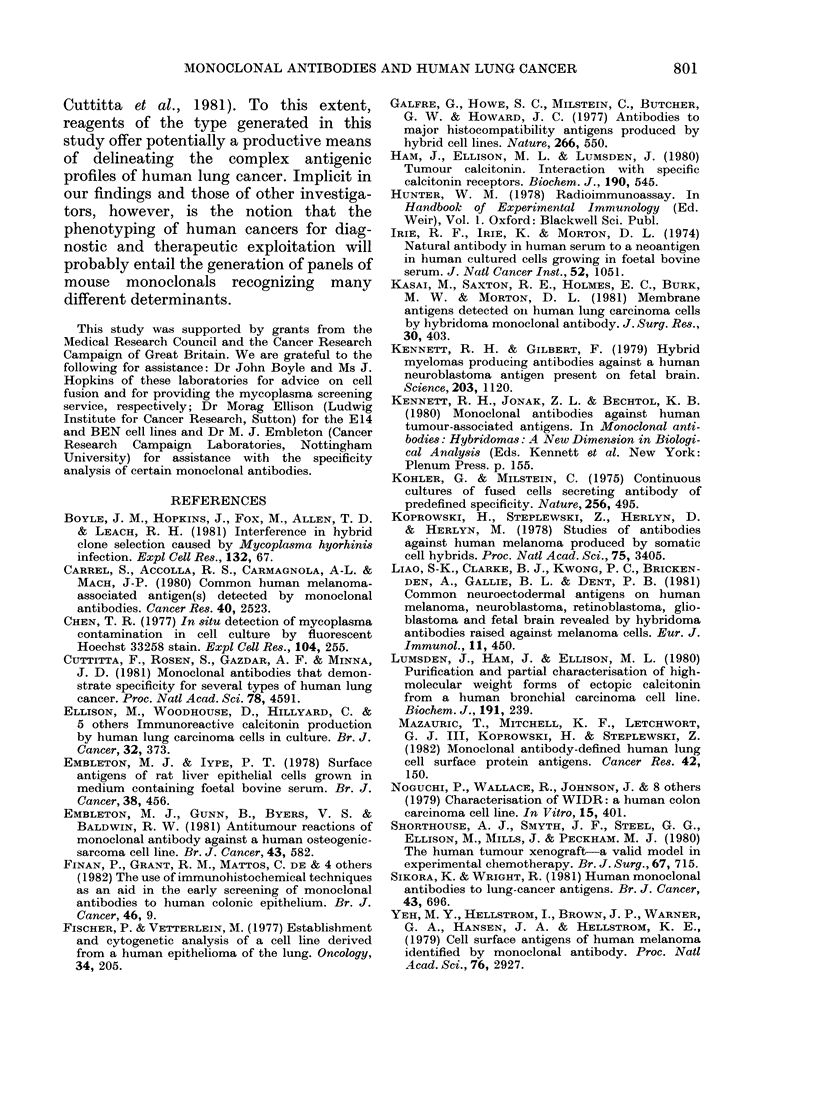# Monoclonal antibodies against two human lung carcinoma cell lines

**DOI:** 10.1038/bjc.1982.272

**Published:** 1982-11

**Authors:** D. T. Brown, M. Moore

## Abstract

Monoclonal antibodies against 2 human lung carcinoma cell lines (E14 and BEN) were prepared by production and cloning of somatic cell hybrids between the murine myeloma NS1, and spleens from E14- and BEN-immune BALB/c mice. Approximately 2000 hybrid culture supernatants were screened for antibody simultaneously against the immunizing cell line and lung fibroblasts (573 Lu) using a radiolabelled Protein A binding assay. Although the vast majority secreted antibodies which recognized species-specific antigens, a few supernatants showed marked differential reactivity against E14 or BEN. These were cloned and subsequently tested against a panel of up to 25 human cell lines originating from different neoplastic and non-neoplastic tissues. Two anti-E14 clones (3E19.8 and 4EAB3.7) displayed preferential activity against lung cancer cell lines, but a low level of reactivity was also detectable with cell lines of different tissue provenance. The antibodies of 3 anti-BEN clones (7B3.5, 7B5.4, 7B17.7) likewise recognized antigens present to a higher density on lung cancer cell lines but were also reactive (to a variable extent for the different clones) with a diversity of other tumour cell lines. The antibodies of 2 further clones were exceptional in so far as one (7BC9.1) reacted only with BEN and WIDR (colorectal cancer) cells, while another (7B24.4) reacted, with apparent exclusivity, against BEN cells. With the exception of the latter, the distinction in antigen expression between many of the cell lines was quantitative rather than qualitative and the emergent picture is one of random expression of individual determinants on several disparate types of cancer cells, rather than restriction to cells of a given morphological type or histogenic derivation.


*Br. J. Cancer* (1982) **46**, 794

### MONOCLONAL ANTIBODIES AGAINST TWO HUMAN LUNG CARCINOMA CELL LINES

D. T. BROWN* AND M. MOORE

*From the Division of Immunology, Paterson Laboratories, Christie Hospital and Holt Radium Institute, Manchester M20 9BX*

Received 28 April 1982 Accepted 10 August 1982

**Summary.—Monoclonal antibodies against 2 human lung carcinoma cell lines (E14 and BEN) were prepared by production and cloning of somatic cell hybrids between the murine myeloma NS1, and spleens from E14- and BEN-immune BALB/c mice. Approximately 2000 hybrid culture supernatants were screened for antibody simultaneously against the immunizing cell line and lung fibroblasts (573 Lu) using a radiolabelled Protein A binding assay. Although the vast majority secreted antibodies which recognized species-specific antigens, a few supernatants showed marked differential reactivity against E14 or BEN. These were cloned and subsequently tested against a panel of up to 25 human cell lines originating from different neoplastic and non-neoplastic tissues. Two anti-E14 clones (3E19.8 and 4EAB3.7) displayed preferential activity against lung cancer cell lines, but a low level of reactivity was also detectable with cell lines of different tissue provenance. The antibodies of 3 anti-BEN clones (7B3.5, 7B5.4, 7B17.7) likewise recognized antigens present to a higher density on lung cancer cell lines but were also reactive (to a variable extent for the different clones) with a diversity of other tumour cell lines. The antibodies of 2 further clones were exceptional in so far as one (7BC9.1) reacted only with BEN and WIDR (colorectal cancer) cells, while another (7B24.4) reacted, with apparent exclusivity, against BEN cells. With the exception of the latter, the distinction in antigen expression between many of the cell lines was quantitative rather than qualitative and the emergent picture is one of random expression of individual determinants on several disparate types of cancer cells, rather than restriction to cells of a given morphological type or histogenic derivation.**

SINCE the description by Kohler & Milstein (1975) of somatic cell-hybridization as a means of generating monoclonal antibodies to cell-surface antigens, a considerable body of data has already accumulated on the expression of such antigens on human tumours. Earlier claims of tissue-site specificity and even of unique tumour specificity for some neoplasms (*e.g.* malignant melanoma, colorectal carcinoma, neuroblastoma (Koprowski *et al.*, 1978; Yeh *et al.*, 1979; Kennett & Gilbert, 1979; Carrel *et al.*, 1980) have more recently given place to reports of greater complexity in antigen distribution, including, for example, apparently random expression not associated with any particular tumour type (Embleton *et al.*, 1981), and cross-reactivity between different tumours (malignant melanoma and brain) of common embryological derivation (Liao *et al.*, 1981).

The evocation of monoclonal antibodies to human carcinoma of lung has received limited attention to date. Sikora & Wright (1981) generated antibodies from interspecies hybrids of hilar and bronchial lymph nodes, with rat or mouse myeloma

* Present address and correspondence: Department of Anatomy, St George's Hospital Medical School, Cranmer Terrace, Tooting, London SW17 0RE.

cells, which were preferentially reactive with lung tumour cell membranes, in comparison with those of normal lung tissue. Other investigators, using established cell lines as immunogens, have developed intraspecies hybrids producing antibodies recognizing antigens of variable distribution on lung and other cancer cells (Kasai *et al.*, 1981; Cuttitta *et al.*, 1981).

In this study the distribution of antigens expressed on cultured lung cancer cells and on tumours of different provenance and histology was investigated by the production of monoclonal antibodies to two well-characterized cell lines (E14 and BEN) originating from squamous cell carcinoma of the bronchus (Fischer & Vetterlein, 1977; Ellison *et al.*, 1975; Ham *et al.*, 1980; Lumsden *et al.*, 1980).

TABLE.—*Human cell lines used as targets in binding assays*

| Tissue origin | Designation in text |
|---|---|
| Lung squamous carcinoma[a] | E14, BEN |
| Lung adenocarcinoma | MOR[b] |
| Lung oat cell carcinoma | FRE*, MAR* |
| Lung carcinoma (unspecified) | A549 |
| Lung carcinoma (fibroblastic) | 573, 618T, 756T |
| Normal lung (fibroblastic) | 573Lu, 618Lu, 756Lu |
| Colorectal carcinoma | WIDR, HT29, HCT8 |
| Liver | Chang |
| Osteosarcoma (fibroblastic) | 791T, 788T |
| Malignant melanoma | RPM1 5966 |
| Burkitt lymphoma | Raji |
| Cervical carcinoma | HeLa |
| Ovarian carcinoma | PA-1 |
| Prostate carcinoma | EB33T |
| Bladder carcinoma | T24 |

[a] All cultures given as epithelial monolayers except where otherwise stated.

[b] Derived from a lung tumour xenograft (Shorthouse *et al.*, 1980).

### METHODS AND MATERIALS

*Cells.*—The parental myeloma line used in these experiments was P3-NS1-1-Ag4-1 (NS1) (Flow Laboratories, Irvine, Scotland). Cells were grown in suspension in DMEM supplemented with 10% foetal bovine serum (FBS) and 1 mM pyruvate (Flow Labs) and subcultured at 2× weekly intervals. Frozen stocks were maintained at −70°C in 90% newborn calf serum (NBCS) and 10% dimethyl sulphoxide (Sigma Chemical Co., U.S.A.). Cells used in fusion experiments were harvested during the logarithmic phase of growth, washed ×2 with serum-free DMEM and routinely checked for aminopterin sensitivity before fusion.

The $IgG_1$ secreting mouse myeloma line P3-X63-Ag8 (X63) was maintained as for NS1, as a source of control murine antibody-containing supernates in the binding assays.

Various human cell lines of different provenance and morphology were used in this study (Table). These were routinely maintained in DMEM plus 10% FBS or NBCS. For passage, cells were disaggregated using 0·1% trypsin (Sigma), 0·02M ethylene diamine tetracetic acid (EDTA) (BDH Chemical Ltd, U.K.) in Hanks' balanced salt solution (HBSS). For immunization and binding assays, cells were harvested mechanically where possible, but if trypsin/EDTA was necessary, cells were allowed to recover 24 h in culture medium in siliconized glass bottles (Repelcote, Hopkins and Williams, U.K.). Freedom from mycoplasma contamination was regularly checked by microscopic examination with the DNA-specific fluorescent Hoechst 33258 stain (Chen, 1977; Boyle *et al.*, 1981).

*Immunization.*—BALB/c mice were immunized with E14 and BEN cells. Two fusions (3E and 4EAB) were carried out with spleens from mice which had received $10^7$ E14 cells in 0·1 complete Freund's adjuvant (CFA) i.p. on Days 1 and 15, followed by a booster i.v. injection of $2 \times 10^6$ E14 cells in 0·1 ml PBS 4 days before fusion. [For the 4EAB fusion, cells of the final i.v. inoculum were precoated with mouse antiserum against the normal lung fibroblast line, 573Lu, in an attempt to mask unwanted specificities (Kennett & Gilbert, 1979)]. It was considered unnecessary to use coated cells in the preliminary immunizations since normally only those antigen-reactive cells which receive a specific stimulus immediately prior to fusion produce hybrids (Kennet *et al.*, 1980). Two further fusions (5E and 6E) were conducted with spleens from (i) mice which received an additional i.p. injection of $10^7$ E14 cells in CFA on Day 32, prior to an i.v. boost, with $2 \times 10^6$ untreated cells on Day 51; and (ii) mice which received only a single i.p. injection on Day 1 and an i.v. boost, also with untreated cells on Day 18. In both instances, fusion was carried out 3 days after the final i.v. immunization.

A single fusion (7B) was performed with spleen cells from mice immunized with BEN

cells, the immunization schedule comprising a single i.p. injection of $10^7$ cells in 0·1 ml (CFA) on Day 1, and a booster i.v. injection of $5 \times 10^6$ uncoated cells on Day 16. Fusion was conducted 3 days later.

*Cell fusion.*—Spleens were removed aseptically and cell suspensions prepared by teasing fragments in DMEM. These cells ($10^8$) were then fused with NS1 cells ($10^7$) using 50% (v/v) polyethylene glycol (PEG), after the basic method of Galfre *et al.* (1977), under conditions capable of generating 3000–5000 hybrids per fusion. To avoid overgrowth of antibody-producing hybrids by non-producing hybrid cells, fused cells were plated at a low density into multiple 96-well tissue culture plates, with extraneous unfused "feeder cells" (spleen cells from mice immune to an irrelevant antigen) to foster hybrid colony development. Supernatants were simultaneously screened for reactivity against the immunizing cell lines, E14 and BEN, and the normal lung fibroblast line, 573Lu, using a $^{125}$I-labelled protein A binding test. Selected positive hybridomas were cloned by limiting dilution.

$^{125}$I *Protein A Binding Assay.*—Protein A (Pharmacia, Sweden) was iodinated by the chloramine T method (Hunter, 1978). Harvested cells ($2 \times 10^5$ cells/25 $\mu$l PBS containing 1% bovine serum albumin (BSA) were admixed with test supernate (50 $\mu$l) in round-bottomed microtitre plates (Flow Laboratories). After incubation at 4°C for 45 min, the cells were washed ×3 by centrifugation and $^{125}$I-labelled protein A (diluted in wash buffer to give an input of approx. $1\text{–}2 \times 10^5$ ct/min) added. Following further incubation at 4°C for 45 min the cells were again washed ×3 and the pellet and washings transferred to LP3 tubes (Luckham Ltd, U.K.) for $\gamma$-counting. Each supernate was assayed in triplicate and the results expressed as mean ct/min minus background values which varied for each target (as determined by control supernates derived from the $IgG_1$ secreting X63 mouse myeloma line) or as a ratio of ct/min bound divided by background values.

More rapid screening for antibodies reactive with adherent cell lines was achieved using a monolayer assay. For this purpose, trypsinized targets ($5 \times 10^4\text{–}5 \times 10^5$ in 200 $\mu$l medium) were added to wells of flat-bottomed microtitre plates (Gibco Europe Ltd), each of which contained a single sterile 6 mm round glass coverslip (Chance-Propper Ltd, U.K.). Confluent monolayers were obtained between 2 and 5 days depending on the input number and these were fixed with glutaraldehyde (0·25% in PBS) (BDH Chemicals Ltd, U.K.) for 10 min at room temperature to improve adherence, washed and maintained at 4°C under PBS containing BSA (for <3 days) before deployment in the binding assay. This was carried out essentially as for the suspension assay except that centrifugation was obviated and coverslips were transferred direct to LP3 tubes for counting. Comparison of data obtained with the same targets in suspension and monolayer assays indicated that they could be used interchangeably. Suspensions were used for specificity analysis and the monolayer modification for screening where large numbers of supernates were involved.

### RESULTS

### *Monoclonal antibodies against E*14

Supernates of 46/485 (9·5%) wells screened from 10 × 96 well microtitre plates of one fusion (3E) contained antibodies reactive with E14 cells, the majority of which were also reactive with 573Lu cells.

Cells generating supernatants with proportionately greater anti-E14 reactivity as well as a number equally reactive with 573Lu cells were cloned. Some hybrids which showed only low reactivity in the primary assays, failed to generate a stable antibody-producing hybrid (presumably on account of instability of the original antibody-producing cells).

Fig. 1 shows the reactivity of different 3E hybridoma lines against various targets in suspension assays. 3E10.2 and 3E22.1 antibodies reacted with 2/3 lung carcinoma cell lines, a colorectal carcinoma cell line (WIDR) as well as the normal 573Lu line, while the 3E11.5 antibody reacted with all cell lines against which it was tested. The 3E14.5 antibody also reacted with all cell lines although binding was greater with the 5 epithelial carcinoma lines (especially E14) than with the lung fibroblast lines. The 3E19.8 antibody reacted most strongly with the carcinoma cell lines of lung origin (again especially

FIG. 1.—Reactivity of 3E hybrid supernatants with various human cell lines. * Significant binding $P < 0 \cdot 05$.

FIG. 2.—Reactivity of 4EAB hybrid supernatants with various human cell lines. * Significant binding $P < 0 \cdot 05$.

with E14 itself), but binding was also detectable against other epithelial cancer cells, lymphoblastoid Raji cells and fibroblast lines. This pattern of reactivity suggested the recognition of the antigen predominantly expressed on most lung cancer cell lines but also present on various other cell lines including some of non-pulmonary origin and possibly also of non-neoplastic phenotype.

In a second fusion (4EAB), supernates of 37/235 wells (16%) contained anti-E14 antibodies. As in fusion 3E, most of these were also reactive against 573Lu cells. However, one supernatant (4EAB3) showed a relatively greater binding to E14. The corresponding hybridoma was subsequently cloned and the antibody tested for reactivity against the larger panel of targets (Fig. 2).

Antibodies secreted by the 4EAB8.2 and 4EAB10.4 clones bound to varying degrees to all the cell lines tested. By contrast, the product of clone 4EAB3.7 showed significant binding with only 4 cell lines, of which 3 were lung carcinomas (E14, BEN, MOR) and the fourth a colorectal carcinoma line (HT29).

Two further fusions (5E and 6E) based upon different immunization protocols from which a total of 695 hybrids were screened failed to generate any antibodies reactive with E14 cells which were not also reactive to varying degrees, with 573Lu.

*Monoclonal antibodies against BEN*

Supernates of 60/286 (42%) and 53/240 (43%) wells screened of one fusion (7B) contained antibodies reactive with BEN and E14 cells respectively, the majority of which were also reactive with 573Lu. However, several supernatants showed a reactivity which suggested the recognition of antigens present at different densities on the 2-types of cell lines. The hybridomas producing these supernatants were cloned and the specificity of the supernatants assayed against all 3 cell lines. Of 28 wells cloned, 24 produced stable hybridoma

FIG. 3.—Reactivity of 7B hybrid supernatants with various human cell lines. * Significant binding $P < 0 \cdot 05$.

lines, of which 16 produced anti-E14 and/or anti-BEN antibodies.

Another group of 96 hybridomas from this fusion were cloned and the supernatants (termed 7BC) screened simultaneously against both lung carcinoma cell lines and against normal lung cells.

While the majority of these were polyspecific (*i.e.* reactive against 573Lu in addition to E14/BEN), or (in one instance) reactive with serum components, one supernate (designated 7BC9) was identified as of potential importance in that it appeared to discriminate between BEN and E14 cells.

Supernatants from several cloned lines resulting from this fusion (7B and 7BC hybrid lines) were tested in suspension for reactivity against the cell line panel. Fig. 3 shows the binding data for 3 monoclonal antibodies which showed a more varied pattern of reactivity. The 7B3.5 antibody reacted with all 5 lung carcinoma lines tested, but with only one of 4 fibroblastic lung lines. It also bound to 1/2 colorectal carcinoma lines (HT29), 2/2 osteosarcoma lines (79IT and 788T) and single lines derived from malignant melanoma (RPMI 5966) and bladder (T24) and cervical (HeLa) carcinomas. Other carcinomas (of ovarian and prostatic origin) and the Raji cell line were negative. The 7B5.4 antibody also bound to all the lung cancer lines as well as 5/7 other epithelial cancers, 2 osteosarcoma cell lines, the malignant melanoma line RPMI 5966 and Raji, but was negative with all 4 fibroblast lines, the ovarian carcinoma PAI and prostatic carcinoma EB33J. The reactivity of the 7B17.7 antibody was more variable. The antigen was present on E14 (but not A549 cells) as well as at low density on the fibroblastic lung lines, an osteosarcoma cell line, 2 other epithelial cancers and the melanoma line.

FIG. 4.—Reactivity of 7B hybrid supernatants with various human cell lines. * Significant binding $P < 0{\cdot}05$.

Two monoclonal antibodies were produced which recognized a target antigen of very restricted distribution. The 7BC9.1 antibody bound only to the immunizing lung carcinoma BEN and at a much lower degree to the colorectal carcinoma cell line, WIDR (Noguchi *et al.*, 1979). Also, the 7B24.4 antibody recognized a determinant expressed at high density only on the immunizing BEN cell line but which was undetectable on 12 other human cell lines tested (Fig. 4). The specificity of these latter antibodies was thus greater than any other generated in the study.

None of the antibodies showing any selectivity for the human cell lines was reactive with sheep or human red blood cells (of whatever major group).

### DISCUSSION

From all fusions with spleens from mice immunized with E14 cells, the frequency of monoclonal antibodies reactive with cell-surface antigens of restricted distribution was low. Widely cross-reacting, species-specific antigens were the immunodominant determinants giving rise to these antibodies, the elicitation of which was largely independent of the immunization protocol. Even coating E14 cells with murine antiserum against the lung fibroblast 573Lu line for the final i.v. injection, a procedure designed to mask the response to unwanted determinants (Kennett & Gilbert, 1979) failed to yield antibodies with more restricted specificity.

Only 2 clones (3E19.8 and 4EAB3.7) of 150 producing anti-E14 antibody appeared to be recognizing antigens predominantly associated with lung carcinoma cells. For the 3E19.8 antibody the distinction was essentially quantitative in so far as, although reactivity with lung cancer cell lines (especially E14) was greatest, a low but significant level of binding was also detectable against lung tumour-derived cells of fibroblast morphology, transferred B cells (Raji), and 2/3 colorectal carcinomas. Greater selectivity was displayed by antibody of the 4EAB3.7 clone. The antigen detected by this antibody was found only on lung carcinoma cell lines (E14, BEN, MOR), on 1/3 colorectal carcinoma lines, but was absent from 15 other human cell types. This virtually excludes the possibility that the antigen is an FBS component incorporated into the membrane of cells grown in medium containing supplementary FBS (Irie *et al.*, 1974; Embleton & Iype, 1978). However, it is possible that the antibody reacts with antigens acquired by some cells as a consequence of *in vitro* passage, rather than with antigens associated with the transformed state. For this reason, prospective testing of antibodies by immunohistology should be used to amplify binding assays against select cell lines (Finan *et al.*, 1982). Even so it appears that this antibody reacts with a determinant expressed ony by certain human carcinoma cells of different origin.

Similar overall, but individually variable patterns of reactivity were observed for clones producing anti-BEN antibodies (7B3.5, 7B5.4, 7B17.7). The target antigens recognized by these antibodies again appear to be present at a higher density on most lung cancer cell lines and a variety of other epithelial, and mesenchymal cancers, but not on fibroblasts. Provisionally, therefore, these antibodies appear to be detecting antigens predominantly associated with the neoplastic state, but further specificity testing is necessary to confirm this. Since the antibodies react with both epithelial and mesenchymal tumour cells it would appear that they are not recognizing differentiation antigens. Also the pattern of reactivity is not what one would expect against polymorphic histocompatibility determinants. Likewise, Forssman antigen and major blood group antigens could be discounted since no reaction with these antibodies was obtained against human or sheep red blood cells.

One monoclonal antibody (7B24.4) appeared to react only with the immunizing cell line, BEN, while another (7BC9.1) reacted with BEN and one other (colorectal cancer) cell line (WIDR). Again, the possibility that these antibodies are detecting antigens expressed adventitiously and in the case of 7B24.4, exclusively by BEN cells as a result of *in vitro* passage cannot be excluded. It is also possible that they are recognizing rare histocompatibility determinants virtually confined to this cell line. Since the antibodies were unreactive toward other lung carcinoma cell lines, the target antigens are clearly not of widespread occurrence but whether this antigen is a truly unique product of the BEN cell line would require much more extensive testing.

Our experience in this study leads us to tentatively conclude that the antigens on human cell lines recognized by monoclonal antibodies raised against them are somewhat randomly distributed and not unequivocally associated with lines derived from tumours of any particular histological type or tissue origin. In particular, evidence that human tumours express antigens common to neoplasms arising in a given tissue as formulated originally for several human cancers, has been conspicuously lacking. However, our data are consistent with more recent reports that antigens—including some of defined molecular weight (Mazauric *et al.*, 1982)—expressed on lung cancer cells may also be shared to a variable degree by non-pulmonary cancers (Kasai *et al.*, 1981;

Cuttitta *et al.*, 1981). To this extent, reagents of the type generated in this study offer potentially a productive means of delineating the complex antigenic profiles of human lung cancer. Implicit in our findings and those of other investigators, however, is the notion that the phenotyping of human cancers for diagnostic and therapeutic exploitation will probably entail the generation of panels of mouse monoclonals recognizing many different determinants.

This study was supported by grants from the Medical Research Council and the Cancer Research Campaign of Great Britain. We are grateful to the following for assistance: Dr John Boyle and Ms J. Hopkins of these laboratories for advice on cell fusion and for providing the mycoplasma screening service, respectively; Dr Morag Ellison (Ludwig Institute for Cancer Research, Sutton) for the E14 and BEN cell lines and Dr M. J. Embleton (Cancer Research Campaign Laboratories, Nottingham University) for assistance with the specificity analysis of certain monoclonal antibodies.

### REFERENCES

BOYLE, J. M., HOPKINS, J., FOX, M., ALLEN, T. D. & LEACH, R. H. (1981) Interference in hybrid clone selection caused by *Mycoplasma hyorhinis* infection. *Expl Cell Res.*, **132**, 67.

CARREL, S., ACCOLLA, R. S., CARMAGNOLA, A-L. & MACH, J-P. (1980) Common human melanoma-associated antigen(s) detected by monoclonal antibodies. *Cancer Res.* **40**, 2523.

CHEN, T. R. (1977) *In situ* detection of mycoplasma contamination in cell culture by fluorescent Hoechst 33258 stain. *Expl Cell Res.*, **104**, 255.

CUTTITTA, F., ROSEN, S., GAZDAR, A. F. & MINNA, J. D. (1981) Monoclonal antibodies that demonstrate specificity for several types of human lung cancer. *Proc. Natl Acad. Sci.* **78**, 4591.

ELLISON, M., WOODHOUSE, D., HILLYARD, C. & 5 others Immunoreactive calcitonin production by human lung carcinoma cells in culture. *Br. J. Cancer*, **32**, 373.

EMBLETON, M. J. & IYPE, P. T. (1978) Surface antigens of rat liver epithelial cells grown in medium containing foetal bovine serum. *Br. J. Cancer*, **38**, 456.

EMBLETON, M. J., GUNN, B., BYERS, V. S. & BALDWIN, R. W. (1981) Antitumour reactions of monoclonal antibody against a human osteogenic-sarcoma cell line. *Br. J. Cancer*, **43**, 582.

FINAN, P., GRANT, R. M., MATTOS, C. DE & 4 others (1982) The use of immunohistochemical techniques as an aid in the early screening of monoclonal antibodies to human colonic epithelium. *Br. J. Cancer*, **46**, 9.

FISCHER, P. & VETTERLEIN, M. (1977) Establishment and cytogenetic analysis of a cell line derived from a human epithelioma of the lung. *Oncology*, **34**, 205.

GALFRE, G., HOWE, S. C., MILSTEIN, C., BUTCHER, G. W. & HOWARD, J. C. (1977) Antibodies to major histocompatibility antigens produced by hybrid cell lines. *Nature*, **266**, 550.

HAM, J., ELLISON, M. L. & LUMSDEN, J. (1980) Tumour calcitonin. Interaction with specific calcitonin receptors. *Biochem. J.*, **190**, 545.

HUNTER, W. M. (1978) Radioimmunoassay. In *Handbook of Experimental Immunology* (Ed. Weir), Vol. 1. Oxford: Blackwell Sci. Publ.

IRIE, R. F., IRIE, K. & MORTON, D. L. (1974) Natural antibody in human serum to a neoantigen in human cultured cells growing in foetal bovine serum. *J. Natl Cancer Inst.*, **52**, 1051.

KASAI, M., SAXTON, R. E., HOLMES, E. C., BURK, M. W. & MORTON, D. L. (1981) Membrane antigens detected on human lung carcinoma cells by hybridoma monoclonal antibody. *J. Surg. Res.*, **30**, 403.

KENNETT, R. H. & GILBERT, F. (1979) Hybrid myelomas producing antibodies against a human neuroblastoma antigen present on fetal brain. *Science*, **203**, 1120.

KENNETT, R. H., JONAK, Z. L. & BECHTOL, K. B. (1980) Monoclonal antibodies against human tumour-associated antigens. In *Monoclonal antibodies: Hybridomas: A New Dimension in Biological Analysis* (Eds. Kennett *et al.* New York: Plenum Press. p. 155.

KOHLER, G. & MILSTEIN, C. (1975) Continuous cultures of fused cells secreting antibody of predefined specificity. *Nature*, **256**, 495.

KOPROWSKI, H., STEPLEWSKI, Z., HERLYN, D. & HERLYN, M. (1978) Studies of antibodies against human melanoma produced by somatic cell hybrids. *Proc. Natl Acad. Sci.*, **75**, 3405.

LIAO, S-K., CLARKE, B. J., KWONG, P. C., BRICKENDEN, A., GALLIE, B. L. & DENT, P. B. (1981) Common neuroectodermal antigens on human melanoma, neuroblastoma, retinoblastoma, glioblastoma and fetal brain revealed by hybridoma antibodies raised against melanoma cells. *Eur. J. Immunol.*, **11**, 450.

LUMSDEN, J., HAM, J. & ELLISON, M. L. (1980) Purification and partial characterisation of high-molecular weight forms of ectopic calcitonin from a human bronchial carcinoma cell line. *Biochem. J.*, **191**, 239.

MAZAURIC, T., MITCHELL, K. F., LETCHWORT, G. J. III, KOPROWSKI, H. & STEPLEWSKI, Z. (1982) Monoclonal antibody-defined human lung cell surface protein antigens. *Cancer Res.* **42**, 150.

NOGUCHI, P., WALLACE, R., JOHNSON, J. & 8 others (1979) Characterisation of WIDR: a human colon carcinoma cell line. *In Vitro*, **15**, 401.

SHORTHOUSE, A. J., SMYTH, J. F., STEEL, G. G., ELLISON, M., MILLS, J. & PECKHAM. M. J. (1980) The human tumour xenograft—a valid model in experimental chemotherapy. *Br. J. Surg.*, **67**, 715.

SIKORA, K. & WRIGHT, R. (1981) Human monoclonal antibodies to lung-cancer antigens. *Br. J. Cancer*, **43**, 696.

YEH, M. Y., HELLSTROM, I., BROWN, J. P., WARNER, G. A., HANSEN, J. A. & HELLSTROM, K. E., (1979) Cell surface antigens of human melanoma identified by monoclonal antibody. *Proc. Natl Acad. Sci.*, **76**, 2927.